# Impaired social brain network for processing dynamic facial expressions in autism spectrum disorders

**DOI:** 10.1186/1471-2202-13-99

**Published:** 2012-08-13

**Authors:** Wataru Sato, Motomi Toichi, Shota Uono, Takanori Kochiyama

**Affiliations:** 1 The Hakubi Project, Primate Research Institute, Kyoto University, Inuyama, Aichi 484-8506, Japan; 2 The Organization for Promoting Developmental Disorder Research, 40 Shogoin-Sannocho, Sakyo-ku, Kyoto 606-8392, Japan; 3 Faculty of Human Health Science, Graduate School of Medicine, Kyoto University, 53 Shogoin-Kawaharacho, Sakyo-ku, Kyoto 606-8507, Japan

**Keywords:** Amygdala, Autism spectrum disorders (ASD), Dynamic facial expression, Fusiform gyrus, Inferior frontal gyrus, Medial prefrontal cortex, Middle temporal gyrus/superior temporal sulcus, Mirror neuron system

## Abstract

**Background:**

Impairment of social interaction via facial expressions represents a core clinical feature of autism spectrum disorders (ASD). However, the neural correlates of this dysfunction remain unidentified. Because this dysfunction is manifested in real-life situations, we hypothesized that the observation of dynamic, compared with static, facial expressions would reveal abnormal brain functioning in individuals with ASD.

We presented dynamic and static facial expressions of fear and happiness to individuals with high-functioning ASD and to age- and sex-matched typically developing controls and recorded their brain activities using functional magnetic resonance imaging (fMRI).

**Result:**

Regional analysis revealed reduced activation of several brain regions in the ASD group compared with controls in response to dynamic versus static facial expressions, including the middle temporal gyrus (MTG), fusiform gyrus, amygdala, medial prefrontal cortex, and inferior frontal gyrus (IFG). Dynamic causal modeling analyses revealed that bi-directional effective connectivity involving the primary visual cortex–MTG–IFG circuit was enhanced in response to dynamic as compared with static facial expressions in the control group. Group comparisons revealed that all these modulatory effects were weaker in the ASD group than in the control group.

**Conclusions:**

These results suggest that weak activity and connectivity of the social brain network underlie the impairment in social interaction involving dynamic facial expressions in individuals with ASD.

## Background

Individuals with autism spectrum disorders (ASD) are characterized primarily by qualitative impairments in social interaction [1]. One of the most evident features of their social impairment involves deficient communication via emotional facial expressions [2]. For example, several previous behavioral studies reported that individuals with ASD exhibited less attention [3], attenuated emotional behaviors [4], and reduced and/or inappropriate facial reactions [5] in response to the facial expressions of other individuals compared with typically developing individuals.

Several neuroimaging studies using functional magnetic resonance imaging (fMRI) and positron emission tomography tested the neural substrates of impaired facial-expression processing in ASD and reported inconsistent findings. Almost all these studies used photos of emotional facial expressions as stimuli and found that individuals with ASD showed abnormal activities in several brain regions, including the posterior superior temporal sulcus (STS) or its adjacent regions such as the middle temporal gyrus (MTG) [6-8], the posterior fusiform gyrus (FG) [7,9-13], amygdala (AMY) [6-8,12], medial prefrontal cortex (MPFC) at around the medial superior frontal gyrus [8,14]**,** and the inferior frontal gyrus (IFG) [9,15,16]. Most of these studies reported hypo activation of these regions [6-11,13-16] (however, see [12]). Substantial neuroimaging and neuropsychological evidence in typically developing individuals has suggested that these brain regions are related to social activities, such as the visual analysis of dynamic aspects of faces involving the STS/MTG [17], the visual analysis of invariant aspects of faces and/or subjective perception of faces involving the FG [18], emotional processing involving the AMY [19], attribution of mental states involving the MPFC [20], and motor mimicry involving the IFG [21]. Based on these data, these regions have been called “social brain” regions [22-28]. Hence, the findings in individuals with ASD appear to account for their impaired processing of emotional facial expressions. However, it must be noted that different studies have reported abnormalities in different parts of the social brain, and thus the results appear to be far from consistent. Furthermore, whether the neural substrates of impaired expression processing in ASD can be traced to reduced activity in any specific brain region and/or to reduced connectivity among the regions, which has been suggested in other lines of ASD research (cf. [29]), remains unknown.

Dynamic facial expressions are more natural and powerful cues in real-life social interaction than are static expressions. From an evolutionary perspective [30-32], human minds are programmed to efficiently process dynamic facial expressions of conspecifics compared with their static expressions, which are artificial signals or products of technology. The importance of the dynamic properties of facial expressions is illustrated by behavioral studies of typically developing individuals. Researchers who observed facial expressions in real situations described rich, dynamic information in emotional facial expressions [31,33]. Several experimental studies have indicated that dynamic facial expressions, as compared with static expressions, induced more evident psychological activities, such as perception (e.g., [34]), emotional reactions (e.g., [35]), and facial mimicry (e.g., [36]). Advantages of using dynamic compared to static facial expressions to induce behavioral reactions have even been shown in newborn infants [37]. Consistent with these behavioral data, several neuroimaging studies with typically developing participants have shown that the social brain regions were more active when viewing dynamic as compared to static facial expressions [38-42]. These regions included the STS/MTG [38-42], FG [38-40], AMY [39,40,42], MPFC [38,39], and IFG [39,40,42].

Nevertheless, few studies have investigated brain activities in response to dynamic facial expressions in individuals with ASD. Impaired social interaction via emotional expression has consistently been shown in individuals with ASD in real situations [3-5], and dynamic, not static, facial expressions would be plausible mediums for such impairments**.** Consistent with this idea, several behavioral studies have demonstrated that impairments in the ability of individuals with ASD to process emotional expressions were more evident in response to dynamic than to static facial expressions (e.g., [43]). Therefore, it is reasonable to assume that neuroimaging studies using dynamic facial expressions would more clearly identify abnormal brain activities in these participants. Pelphrey et al. [44] tested this issue by presenting dynamic and static facial expressions depicting anger, fear, and neutral emotions to a group of individuals with ASD and to typically developing controls. The researchers found that the observation of dynamic facial expressions elicited less activation in the ASD group as compared with the control group in several social brain regions including the STS/MTG, FG, AMY, and MPFC. These data suggest that this reduced brain activation in response to dynamic facial expressions reflects the neural basis of impaired facial expression processing in individuals with ASD. However, this study did not reveal clear IFG activity in either the ASD or the control group. This issue could be critical because the IFG has recently received considerable interest in the neuroscientific literature on ASD. Indeed, it has been suggested that the IFG contains specific neuronal populations, known as “mirror neurons,” that discharge both when observing and when executing specific actions (for reviews, see [45,46]). In the context of behavioral data indicating abnormal mimicking in ASD (e.g., [47]), some researchers have proposed that IFG dysfunction may constitute a fundamental deficit in ASD [48-50]. We reasoned that we could clarify this issue by using dynamic facial expression stimuli that were shown to effectively activate the IFG in typically developing individuals [40]. We hypothesized that the observation of dynamic, compared with static, facial expressions would clearly reveal hypo activation of social brain regions (i.e., STS/MTG, FG, AMY, MPFC, and IFG) in individuals with ASD.

Furthermore, the functional network patterns of the social brain regions for processing dynamic facial expressions in both typically developing individuals and those with ASD remain unknown. A previous study tested the effective connectivity in typically developing control and ASD groups using dynamic facial expressions as stimuli and found differential patterns of effective connectivity between groups [51]. However, because that study focused on the effects of tasks, the functional network underpinning the processing of dynamic facial expressions *per se* remains to be tested. Among the components of the social brain, converging data from anatomical and theoretical studies suggest that the STS/MTG and IFG constitute the circuit. Several anatomical studies, including histological examinations in humans [52,53] and non-human primates [54,55], as well as diffusion tensor imaging in humans [56-58] and non-human primates [57], indicated that the STS/MTG and IFG are directly connected. Some researchers have proposed that this circuit serves an important function in social interaction as the mirror neuron system (MNS) in typically developing individuals and is impaired in individuals with ASD [50,59,60]. However, this idea remains to be empirically tested. Based on these data, we hypothesized that observation of dynamic versus static facial expressions would enhance the functional couplings of the neural networks including the STS/MTG and IFG of typically developing individuals and that reductions would be found in the same functional neural networks of individuals with ASD.

In the present fMRI study, we examined the brain activities of a group of high-functioning individuals with ASD and age- and sex-matched typically developing controls while they viewed dynamic and static facial expressions. The stimuli used to depict dynamic facial expressions were shown to activate the social brain regions, including the IFG, in typically developing participants [40]. The stimuli were also found to sufficiently represent natural changes in facial expressions [61] and to effectively induce subjective emotion [35] and facial mimicry [36] in typically developing individuals. We prepared facial expressions with both negative (fearful) and positive (happy) emotional valences. The participants were asked to discriminate the sex of the presented faces to ensure that they were attending to the stimuli and to prevent their explicit processing of the emotional expressions. By comparing the brain activities under dynamic versus static facial expression conditions, we identified the regions involved in the processing of dynamic facial expressions. Furthermore, to investigate effective connectivity, we conducted dynamic causal modeling (DCM).

## Results

### Behavioral performance

The correct response percentage of the sex-discrimination task was comparable across groups: dynamic fear (control: *M* = 98.4, *SD* = 1.1; ASD: *M* = 92.4, *SD* = 5.3), dynamic happiness (control: *M* = 98.7, *SD* = 1.0; ASD: *M* = 93.4, *SD* = 5.3), static fear (control: *M* = 98.7, *SD* = 1.0; ASD: *M* = 93.9, *SD* = 4.3) and static happiness (control: *M* = 97.4, *SD* = 1.3; ASD: *M* = 93.8, *SD* = 3.8). A three-way repeated-measures analysis of variance (ANOVA) using group, presentation condition, and emotion as factors on the correct response percentage showed no significant main effects or interactions.

Correct response reaction times (RTs) were also comparable across groups: dynamic fear (control: *M* = 231.3, *SD* = 25.5; ASD: *M* = 242.0, *SD* = 52.7), dynamic happiness (control: *M* = 237.8, *SD* = 28.6; ASD: *M* = 205.7, *SD* = 53.0), static fear (control: *M* = 183.0, *SD* = 21.0; ASD: *M* = 186.4, *SD* = 45.5) and static happiness (control: *M* = 182.0, *SD* = 22.1; ASD: *M* = 201.3, *SD* = 41.2). An ANOVA with the same design as described above on the correct RTs showed only a significant main effect of presentation condition, indicating longer RTs in response to dynamic than to static presentations (*F*(1,23) = 13.96, *p* < .005).

In summary, behavioral performance data revealed no significant effects related to group.

### Regional brain activity

**Figure 1:**
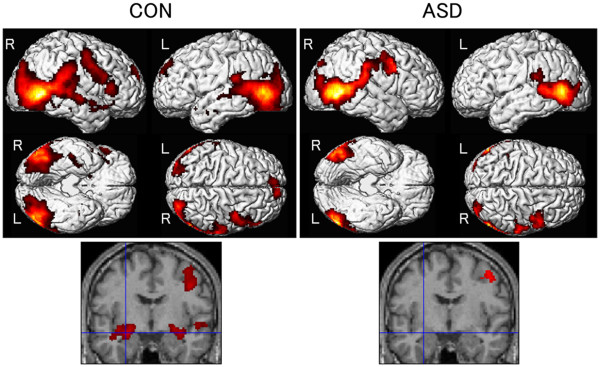
**Statistical parametric maps showing significant brain activation for dynamic versus static facial expressions.** The control (CON) and autism spectrum disorders (ASD) groups are shown in the left and right panels, respectively. The areas of activation are rendered on spatially normalized brains (upper) and overlaid on the normalized anatomical MRI of one of the participants at the coronal section showing amygdala activation (lower). The cross hairs in the lower panels are centered on the activation focus of the left amygdala in the control group (*x* -26, *y* -6, *z* -16; *t* = 4.65; cluster size = 5368 mm^3^). An extent threshold of *p* < .05, corrected for multiple comparisons, with a height threshold of *p* < .01 (uncorrected) were used. L = Left hemisphere; R = Right hemisphere.

We tested regional brain activity using the three-way repeated-measures ANOVA model with group, presentation condition, and emotion as factors (Additional file 1: Figure S1). Initially, the simple main effect of presentation condition, contrasting dynamic and static presentations, was tested for each group (Table 1; Figure 1). For the control group, broad ranges of bilateral posterior regions, which included activation of the MTG and FG, were detected as areas of significant activation. Significant activation was also observed in the bilateral AMY, bilateral MPFC, and right IFG. For the ASD group, bilateral activation of the posterior regions was found to be significant, although its size was smaller than that of the control group. No other areas showed significant activation, including such social brain regions as the AMY, MPFC, and IFG.

**Table 1:** Brain regions showing significant activation for dynamic versus static facial expressions

**Brain region**	**BA**	**Control**					**ASD**				
		**Coordinates**			** *T* -value**	**Cluster**	**Coordinates**			** *T* -value**	**Cluster**
		** *x* **	** *y* **	** *z* **		**size (mm**^**3**^**)**	** *x* **	** *y* **	** *z* **		**size (mm**^**3**^**)**
R. middle occipital gyrus	19	34	-84	8	7.12	77624	46	-78	0	6.35	27336
R. middle temporal gyrus	37	52	-62	0	17.60		52	-64	-2	9.29	
R. middle temporal gyrus	37	40	-60	-12	6.92						
R. middle temporal gyrus	21	50	-36	6	6.05		48	-48	6	6.21	
R. temporal pole	38	54	6	-12	3.42						
R. supra marginal gyrus	48	54	-28	28	5.75		60	-24	38	3.92	
R. precentral gyrus	6	40	-2	50	4.59		48	-6	46	3.42	
R. inferior frontal gyrus	45	56	28	10	4.47						
R. middle frontal gyrus	9	52	8	36	4.59		46	8	36	3.73	
R. hippocampus	-	32	-10	-16	4.27						
R. amygdala	-	28	-8	-12	4.54						
R. medial superior frontal gyrus	10	6	64	22	4.50	4720					
L. medial superior frontal gyrus	10	-14	50	18	4.76						
L. middle occipital gyrus	19	-48	-78	0	13.45	47592	-52	-72	2	9.28	16928
L. middle temporal gyrus	21	-56	-50	10	6.62		-58	-54	2	4.55	
L. middle temporal gyrus	22	-52	-18	0	3.19						
L. superior temporal gyrus	42	-62	-32	20	3.52						
L. fusiform gyrus	37	-42	-58	-16	4.86						
L. supra marginal gyrus	48	-46	-32	22	4.07						
L. amygdala	-	-26	-6	-16	4.65	5368					

The coordinates of the foci of activation in Montreal Neurological Institute space, the T-values, and the cluster sizes for the control and autism spectrum disorders (ASD) groups are shown in the left and right parts, respectively.The extent threshold of p < .05, corrected for multiple comparisons, with the height threshold of p < .01 (uncorrected) were used.

**Figure 2:**
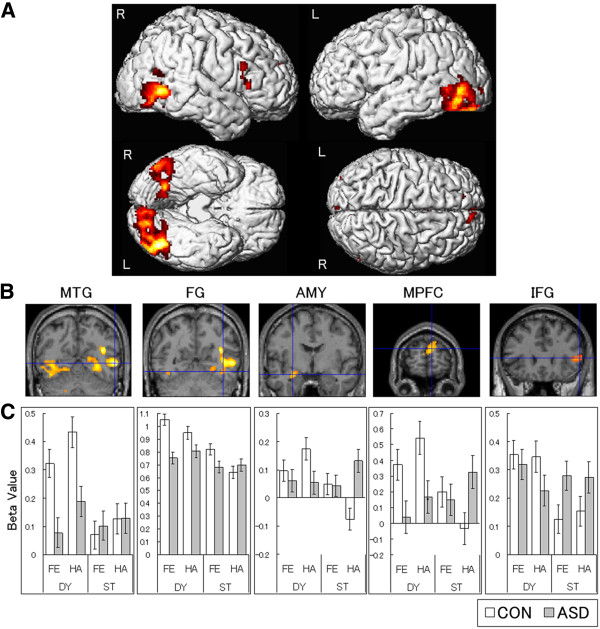
**Brain activation for the significant interaction between group and presentation condition.** Weaker activation was found in the autism spectrum disorders (ASD) group than in the control (CON) group for dynamic (DY) versus static (ST) expressions. **A**. Statistical parametric maps rendered on spatially normalized brains. A height threshold of *p* < .01 (uncorrected) was used without extent threshold restriction for display purposes. L = Left hemisphere; R = Right hemisphere. **B**. Statistical parametric maps of representative brain regions overlaid on the normalized anatomical MRI of one of the participants in this study. From left to right, the activation of the middle temporal gyrus (MTG; *x* 52, *y* -62, *z* 0; *t* = 5.08), fusiform gyrus (FG; *x* 40, *y* -58, *z* -14; *t* = 3.00), amygdala (AMY; *x* -28, *y* -4, *z* -18; *t* = 2.89), medial prefrontal cortex (MPFC; *x* 8, *y* 66, *z* 20; *t* = 3.87), and inferior frontal gyrus (IFG; *x* 48, *y* 26, *z* 8; *t* = 3.06) is shown. The statistical thresholds are the same as above. **C**. Mean parameter estimates (± *SE*) of brain regions corresponding to the above overlaid MRIs. The data were extracted at the sites of peaks. FE = Fear; HA = Happiness.

Then, a planned contrast of the interaction between group and presentation condition was conducted, testing for reduced activation in the ASD as compared with the control group under dynamic versus static conditions (Table 2; Figure 2). The bilateral posterior regions, including the activation foci in the MTG in the right hemisphere and the FG in both hemispheres, were significantly activated. Significant activation was also found in the left AMY, bilateral MPFC, and right IFG. No significant activation was observed in any other region.

**Table 2:** Brain regions showing significant interactions between group and presentation condition

**Brain region**	**BA**	**Coordinates**			** *T* -value**	**Cluster**
		** *x* **	** *y* **	** *z* **		**size (mm**^**3**^**)**
R. inferior occipital gyrus	19	42	-78	-16	3.04	9192
R. inferior temporal gyrus	19	48	-70	-8	3.79	
R. middle temporal gyrus	37	52	-62	0	5.08	
R. fusiform gyrus	37	40	-58	-14	3.00	
R. inferior frontal gyrus	45	48	26	8	3.06	840
R. medial superior frontal gyrus	10	8	66	20	3.87	1864
L. medial superior frontal gyrus	10	-14	50	18	4.33	
L. lingual gyrus	18	-16	-86	-12	3.55	21616
L. inferior occipital gyrus	18	-30	-88	-22	4.27	
L. middle occipital gyrus	19	-48	-80	0	5.42	
L. fusiform gyrus	37	-42	-60	-16	3.08	
L. amygdala	-	-28	-4	-18	2.89	520

The coordinates of the foci of activation in Montreal Neurological Institute space, their *T*-values, and the cluster sizes are shown.

The extent threshold of *p* < .05, corrected for multiple comparisons, with the height threshold of *p* < .01 (uncorrected) were used.

We also conducted exploratory analyses for other interactions related to the group factor in the whole brain, but found no significant results.

### DCM

**Figure 3:**
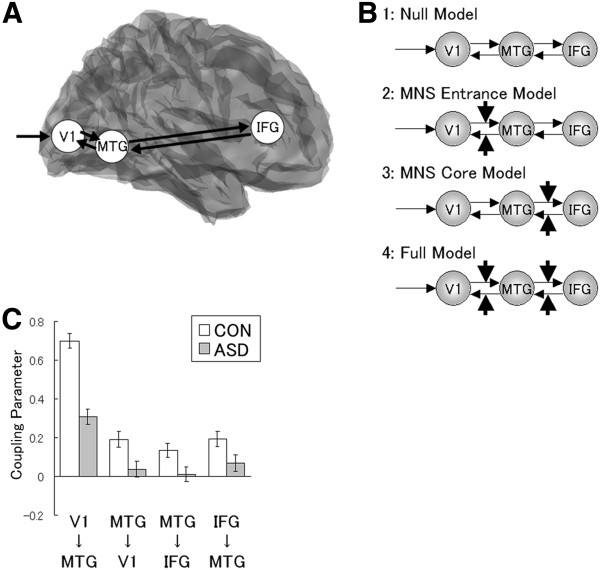
**Models and results of dynamic causal modeling (DCM) regarding the mirror neuron system (MNS). A**. Analyzed brain regions rendered on the spatially normalized brain. V1 = Primary visual cortex; MTG = Middle temporal gyrus; IFG = Inferior frontal gyrus. **B**. Analyzed models. Thin arrows indicate intrinsic connections between brain regions. Bold arrows indicate the modulatory effects of dynamic presentation. **C**. Mean coupling parameters (± *SE*) for the control (CON) and autism spectrum disorders (ASD) groups. Statistical comparisons showed that all parameters were significantly weaker in the ASD than in the control group (*t*-test, *p* < .05).

DCM analyses were conducted to test the MNS network for each group. Bi-directional (forward and backward) intrinsic connections were constructed between the primary visual cortex (V1) and MTG and between the MTG and IFG (Figure 3a). The modulatory effect of dynamic presentation was modeled to modulate each of these bi-directional connections. Based on the locations of the modulatory effects, we constructed the following four models (Figure 3b): (1) the null model, with no modulatory effect; (2) the MNS-entrance modulation model, with modulatory effects on the V1–MTG connections; (3) the MNS-core modulation model, with modulatory effects on the MTG–IFG connections; and (4) the full model, with modulatory effects on both the V1–MTG and MTG–IFG connections. The exceedance probability of the Bayesian model selection (BMS) indicated that the full model was the most likely for both groups (Table 3).

**Table 3:** Summary of the results of Bayesian model selection (BMS) and Bayesian model averaging (BMA)

**Model**	**BMS: Posterior family**		**BMA: Mean (± *SD* ) number of selected**			
	**exceedance probabilities**		**models in the Occam's window**			
	**Control**	**ASD**	**Control**		**ASD**	
1: Null Model	0.00	0.01	0.0	(0.0)	0.3	(0.5)
2: MNS Entrance Model	0.31	0.27	1.0	(0.0)	1.0	(0.0)
3: MNS Core Model	0.00	0.02	0.0	(0.0)	0.3	(0.5)
4: Full Model	0.69	0.70	1.0	(0.0)	1.0	(0.0)

To test group differences in coupling parameters, Bayesian model averaging (BMA) analysis was conducted (Table 3), and the resultant posterior means of modulatory effect parameters (Figure 3c) were analyzed. First, to test for differences from zero, one-sample *t*-tests were conducted for each group. The results showed that the facilitative modulatory effects of dynamic presentation were significant among members of the control group for all bi-directional connections between the V1 and MTG and the MTG and IFG (*t*(12) > 3.76; *p* < .005). Significant facilitative modulatory effects of dynamic presentation were found for the connection from the V1 to the MTG (*t*(11) = 2.73; *p* < .05) but not for any other connections (*t*(11) < 1.20; *p* > .1) in the ASD group. To test for differences between groups, two-sample *t*-tests were conducted. The results showed reduced modulatory effects under the dynamic condition with respect to all connections in the ASD group as compared with the control group (*t*(23) > 1.91; *p* < .05).

## Discussion

### Regional brain activity

Our results regarding regional brain activity in the control group showed that observation of dynamic facial expressions was associated with greater activation than observation of static facial expressions in distributed brain regions including the MTG, FG, AMY, MPFC, and IFG. The activation of these regions is consistent with the findings of previous studies (e.g., [40]). All of these brain regions have been proposed to constitute the social brain network (e.g., [28]); our results confirm that the presentations of dynamic versus static facial expressions are appropriate for activating the social brain networks of typically developing individuals.

More importantly, the group comparison results showed that these social brain regions were less activated in response to dynamic than to static facial expressions in the ASD compared with the control group. Because the participants in the ASD group had no symptoms other than social impairment and repetitive traits, these results can be attributed to the core deficits of ASD. The reduced activation of the social brain regions in individuals with ASD in response to dynamic facial expressions is consistent with the findings of a previous study [44]. Because group differences in IFG activities were not reported in the previous study, the current study is the first to provide evidence that functional abnormality in this region is related to the impaired processing of dynamic facial expressions in ASD. We consider the possibility that some methodological differences may account for the disparity in the results. For example, the stimuli depicting dynamic facial expressions in the present study reflected more rapid changes than did those used in the study conducted by Pelphrey et al. [44]. A previous behavioral study reported that the speed at which dynamic facial expressions changed influenced the recognition of natural facial expressions and suggests that the speed used in the present study was preferable for natural dynamic facial expressions [61]. Because several anatomical studies have reported single-cell and/or population level structural abnormalities in the social brain regions (i.e., STS/MTG [62-64], FG [65,66], AMY [67,68], MPFC [64,69], and IFG [62,64,70]) it is plausible that these regions reflect characteristics of abnormal brain functioning in ASD. Because dynamic facial expressions are realistic mediums for social interaction, our results suggest that the weak activation in these social brain regions is related to the real-life impairments in communication via facial expressions experienced by individuals with ASD.

Previous neuroimaging studies of typically developing participants (e.g., [71-73]; for reviews, see [17,18]) have shown that the STS/MTG is involved in visual analyses of the dynamic or changeable aspects of faces. Previous neuroimaging studies also showed that observation of dynamic point-light displays of human actions activated the STS/MTG in typically developing individuals but not in those with ASD [74,75]. Consistent with these neuroscientific data, several behavioral studies have reported that individuals with ASD showed impaired perception of dynamic human actions [76-80]. In their review of behavioral and neuroscientific studies, Dakin and Frith [81] proposed that individuals with ASD experience impairment in the perception of human actions and that this impairment appears to be related to dysfunction in the STS/MTG. Together with these data, our results suggest that reduced STS/MTG activation is involved in impaired visual analyses of the dynamic aspects of emotional facial expressions experienced by those with ASD.

In contrast, the FG has been shown to relate to the visual analyses of invariant aspects of faces and/or the subjective perception of faces in typically developing participants (e.g., [72,82]; for a review, see [18]). Several previous neuroimaging studies in individuals with ASD have also reported reduced FG activation in processes involved in basic visual discrimination of faces versus non-faces [83-85]. Together with these data, our results suggest that the dynamic presentations of facial expressions enhance the visual analyses or perception of faces in typically developing individuals but not in individuals with ASD.

The AMY has been shown to be involved in emotional processing of typically developing participants while they view dynamic facial expressions [86]. A previous neuroimaging study reported consistent changes in the AMY activities of typically developing controls but not of those with ASD as a function of the intensity of the emotional facial expressions depicted in photos, suggesting abnormal emotional processing in the AMY of individuals with ASD [8]. Several lesion studies in animals have also indicated that damage to the AMY induced abnormal emotional reactions to the emotional expressions of other individuals (e.g., [87]), which have been likened to the socioemotional impairments in ASD [88]. Consistent with these neuroscientific data, a previous behavioral study reported that individuals with ASD did not show higher autonomic and behavioral responses to distressed than to neutral dynamic expressions, although typically developing controls did show such responses [4]. Combined with these data, our results suggest that reduced AMY activation is involved in the impaired emotional reactions to dynamic facial expressions shown by individuals with ASD.

The MPFC has been shown to be activated when participants attributed mental states to others (i.e., mentalizing or theory of mind; e.g., [89]; for a review, see [20]). The ability to mentalize has been proposed as a the specific characteristic that has emerged over the course of human evolution [90] and as constituting a crucial social deficit in ASD [91]. The reduced MPFC activation in mentalizing tasks among individuals with ASD compared with typically developing individuals has also been shown in previous neuroimaging studies [14,92,93]. Our results showing that this region was active in response to dynamic facial expressions among those in the control group suggest that typically developing individuals automatically try to read others’ mental states in real-life social interaction. Furthermore, our results showing group differences in the activities in this region suggest that such automatic mentalizing is relatively less pronounced in those with ASD.

Several previous neuroimaging studies involving typically developing participants have reported greater IFG activation not only when participants passively observed dynamic versus static facial actions [39,40,42,94,95], but also when participants imitated the dynamic facial expressions that they were viewing than compared with when they passively viewed these stimuli [96,97]. This finding is consistent with theories proposing that the IFG contains mirror neurons [45,46], which are activated in response to both the observation and the execution of facial expressions. Previous neuroimaging [16] and magnetoencephalographic [98] studies have consistently indicated that the imitation of facial actions while viewing static facial stimuli induced less activation in the IFG in the ASD than in the control group. Together with these data, our results suggest that the reduced IFG activation in individuals with ASD in response to dynamic facial expressions is related to deficits in automatic facial mimicry in ASD.

It is interesting to note that visual inspection of IFG activities (Figure 2) indicates that the ASD group participants showed clear IFG activation against the resting condition, although the differences between dynamic and static conditions were smaller than those in the control group. Consistent with these data, previous behavioral studies reported that individuals with ASD did not lack facial reactions to the emotional facial expressions of other individuals but instead reacted to the facial expressions differently from the ways in which typically developing individuals reacted [5,99-101]. Collectively, our results suggest that the activation patterns of the mirror neurons in the IFG in individuals with ASD may be altered, perhaps producing abnormal facial mimicry during social interaction involving facial expressions.

### Effective connectivity

Our results regarding the DCM in the control group showed that observation of dynamic compared with static facial expressions enhanced effective connectivity of the MNS network connecting the V1, MTG, and IFG. These results provide a mechanistic account of the enhanced activities manifested by sets of brain regions in response to dynamic facial expressions by construing them as a positively connected circuit. For example, the STS/MTG is more active in response to dynamic than to static faces because the inputs from the V1 through the feed forward connection and the inputs from the IFG through the feedback connection are enhanced. The result also provides suggestions for information flow in the neural processing of dynamic facial expressions: When we observe dynamic facial expressions, the visual information processed through the V1 and STS/MTG is transmitted to the motor processing area in the IFG; then, the motor representation in the IFG modulates visual decoding in the STS/MTG, which then modulates basic visual processing in the V1. These systematic views are consistent with previous theoretical proposals that these brain regions constitute the functional network of the MNS and/or social brain network (e.g., [59]). To our knowledge, this is the first evidence that dynamic facial expressions enhance not only regional brain activities but also effective connectivity among these regions.

More interestingly, our results revealed weaker modulatory effects of dynamic facial expressions on the MNS connections in the ASD group than in the control group. As in the case of the control group, our results provide a mechanistic account of the relatively weak activities of the social brain regions for processing dynamic facial expressions in individuals with ASD: In these individuals, positive connectivity among the regions is weak. For example, STS/MTG activation induced by dynamic versus static facial expressions is reduced because feed forward inputs from the V1 and feedback inputs from the IFG are weaker than those in typically developing individuals. The effect of weak neural connectivity in ASD has been theoretically proposed in several previous studies (e.g., [102]). Previous empirical studies have also reported that individuals with ASD showed reduced functional connectivity while engaging in social tasks, such as expression recognition [51,103], face perception [104,105], mentalizing [92], and other non-social cognitive tasks [106-110]. Our results extend the literature by providing the first evidence that effective connectivity modulation of the social brain network for processing of dynamic facial expressions is reduced in ASD.

Our results showed reduced modulatory effects in both the core (MTG–IFG) and the entrance (V1–MTG) connections of the MNS in the ASD group. These results provide insights into the loci of abnormalities in the social brain networks of those with ASD. As mentioned above, several previous studies have found abnormal activities in the social brain regions of individuals with ASD (e.g., [16]). These data suggest the existence of problems in the core parts of the social brain network in ASD. However, some other studies have reported abnormal activities in the early visual cortices in individuals with ASD (e.g., [111]; for a review, see [112]), suggesting that problems begin before the social brain is involved. Our results allow reconciliation of these lines of research by indicating functional problems at both the entrance and the core of the social brain network among those with ASD.

Our results provide unique explanations and predictions of the behaviors of typically developing individuals and of those with ASD. For example, a previous behavioral study among typically developing individuals showed that intentional facial mimicking facilitated the recognition of dynamic facial expressions [113]. Our results explain this finding by indicating that one’s own facial motor commands related to IFG activation facilitate the visual analyses of others’ facial expressions that are related to MTG activation. Such an idea provides the basis for predicting that the facilitative effect of facial mimicry on expression recognition may be impaired in individuals with ASD.

### Implications, limitations, and future directions

Our results showing the group differences in the functioning of the social brain network in response to dynamic versus static facial expressions have practical implications for experimental studies on ASD. Several behavioral and neuroscientific studies have previously used static emotional facial expressions as stimuli to investigate abnormalities in the processing of emotional expressions in individuals with ASD and have produced inconsistent findings. Based on our results, we propose that the presentations of dynamic facial expressions are more appropriate than the presentations of static expressions for revealing abnormalities in social interaction among those with ASD. Consistent with this idea, some pioneering behavioral studies have found that dynamic presentations of facial stimuli revealed abnormal behavioral patterns characterizing the social interaction of individuals with ASD; these results have not been observed in studies using static presentations. For example, Uono et al. [43] reported that experiments using dynamic facial expressions as stimuli revealed the facilitative effect of emotional expression on automatic gaze-triggered attentional shifts in typically developing individuals and the impairment in this regard among individuals with ASD, although such effects were not found in response to static presentations [114]. We expect that further studies using dynamic facial expressions as stimuli will provide pronounced evidence of the cognitive mechanisms and neural substrates underlying the social impairments of ASD.

Some limitations of this study should be acknowledged. First, the contrast between dynamic emotional and dynamic neutral expressions remains untested. Such a contrast would allow us to discriminate between the effects of facial motion and those of the emotional messages conveyed by dynamic facial expressions. This issue could be intriguing because inconsistent findings have been reported in studies with typically developing individuals regarding social brain activation patterns for dynamic emotional versus dynamic neutral faces (e.g., [39,115,116]). Regarding this issue, Pelphrey et al. [44] measured brain activation in response to dynamic neutral faces, which were derived from identity morphing, and static neutral faces in ASD and typically developing control groups. They found no significant interaction between group (ASD vs. control) and presentation condition (dynamic neutral vs. static neutral) in the activation of the AMY, FG, or STS/MTG. These results suggest that weaker activation of these regions induced by dynamic facial expressions in ASD might not be accounted for by facial motion *per se*. However, this question remains unresolved for the activities of other social brain regions (e.g., the IFG), and further investigation on dynamic neutral faces is an important matter for future research.

Second, we tested only fearful and happy facial expressions. Hence, the effects of dynamic presentations of other emotions on individuals with ASD remain to be examined. Observation of dynamic facial expressions depicting other emotions may reveal abnormal activities in other brain regions among those with ASD. For example, some previous neuroimaging studies with typically developing participants have reported that the observation of dynamic and/or static disgusted facial expressions activated brain regions that were not activated in the present study, including the basal ganglia and insula (e.g., [117,118]; for a review, see [19]). A previous neuroimaging study showed that the observation of photos depicting disgusted facial expression induced less activation in these brain regions in the ASD than in the control group [15], although such a group difference was not evident in another study [13]. We speculate that the observation of dynamic versus static facial expressions of disgust may provide clear evidence of abnormal activities of these brain regions in individuals with ASD.

Third, our study did not record eye movements during participants’ observations of dynamic and static facial expressions, although a previous neuroimaging study suggested that an abnormal fixation pattern on faces reduced FG activation in individuals with ASD [12]. This issue may be relevant because we presented stimuli for 1500 ms to depict dynamic aspects of facial expressions, which is long enough for the participants to make eye movements. To reduce the effect of eye movements, we instructed participants to fixate on a point between the eyes (the center of the screen). Some previous studies [119,120] using the same instruction reported that the FG of individuals with ASD showed normal activation in response to faces. Accordingly, our fMRI results (Figure 2) demonstrated that FG activities in response to static facial expressions were comparable across the ASD and control groups. These data may rule out the possibility that the abnormal fixation pattern on faces would account for the lower levels of brain activation in individuals with ASD. However, such speculation should be verified in future studies recording eye movements during the processing of dynamic facial expressions.

Fourth, our functional coupling analyses were restricted to a part of the social brain network because DCM was designed to test specific hypotheses rather than to act as an exploratory technique [121,122]. Currently, knowledge about the anatomical and functional connections among all social brain regions remains lacking. It is plausible that the MNS is a sub-component in a more widespread network. For example, the AMY may directly modulate the activities of the MTG and IFG or may exert a bilinear modulatory effect on the connection between these regions. Further studies regarding anatomical and functional connectivity are necessary to elucidate the social brain network and related impairments in ASD.

## Conclusions

In summary, our results showed that activation of several brain regions (i.e., MTG, FG, AMY, MPFC, and IFG) in response to dynamic versus static expressions was weaker in the ASD than in the control group. The results also revealed that the modulatory effects of dynamic facial expressions on bi-directional effective connectivity in the V1–MTG–IFG circuit were weaker in the ASD than in the control group. These data suggest that weak activity and connectivity of the social brain network for processing dynamic facial expressions underlie the impairments demonstrated by individuals with ASD in real-life social interaction.

## Methods

### Participants

The ASD group comprised 12 adults (1 female, 11 males; age, *M* = 27.5, *SD* = 7.6). Although an additional male candidate actually participated, his data were not analyzed due to large motion artifacts (>3 mm). The group consisted of eight males with Asperger’s disorder and four (1 female, 3 males) with pervasive developmental disorder not otherwise specified (PDD-NOS). As defined in the Diagnostic and Statistical Manual-Fourth Edition-Text Revision (DSM-IV-TR)[1], PDD-NOS includes heterogeneous subtypes of ASD, ranging from so-called atypical autism to a subgroup with symptoms milder than Asperger’s disorder (i.e., satisfying fewer diagnostic criteria than required for a diagnosis of Asperger’s disorder). In this study, only high-functioning PDD-NOS participants with milder symptoms than those associated with Asperger’s disorder were included. Neurological and psychiatric problems other than those associated with ASD were ruled out. Participants were not taking medication. Therefore, all participants in the ASD group had only the core deficits of ASD (i.e., social impairments and repetitive traits).

The diagnosis was made using DSM-IV-TR by a stringent procedure in which every item of the ASD diagnostic criteria was investigated in interviews with the participants and their parents (and professionals who helped them, if any) by two psychiatrists with expertise in developmental disorders. Only participants who met at least one of the four social impairment items (i.e., impairment in nonverbal communication including lack of joint attention, sharing interest, relationship with peers, and emotional and interpersonal mutuality) without satisfying any items of the criteria of autistic disorder, such as language delay, were included. Comprehensive interviews were administered in order to obtain information about the participants’ developmental histories for diagnostic purposes.

For 10 individuals among the ASD group, the level of symptom severity was quantitatively assessed using the Japanese version of Childhood Autism Rating Scale (CARS) [123] administered by a psychiatrist with expertise in developmental disorders. The CARS is one of the most widely used scales to evaluate the degree of ASD [124]. The CARS scores in the ASD group (*M* = 21.1, *SD* = 1.7) were comparable to those in previous studies with individuals with Asperger’s disorder [124] and individuals with Asperger’s disorder and Asperger type PDD-NOS [125] (*t*-test, *p* > .1). These data support that the symptoms were severe enough in the ASD group.

Full-scale intelligence quotients (IQs), measured by the Wechsler Adult Intelligence Scale-Revised (WAIS-R), of all participants in the ASD group fell within the normal range (full-scale IQ: *M* = 113.1, *SD* = 12.5; verbal IQ: *M* = 117.3, *SD* = 10.8; performance IQ: *M* = 106.3, *SD* = 14.9).

The control group comprised 13 adults (1 female, 12 males; age, *M* = 24.3, *SD* = 3.4). They had no neurological or psychiatric problems. They were recruited through advertisements and were matched with the ASD group for age and sex. The full-scale IQs, measured by the WAIS-R, of all control participants also fell within the normal range (full-scale IQ: *M* = 126.3, *SD* = 6.1; verbal IQ: *M* = 128.1, *SD* = 7.2; performance IQ: *M* = 118.8, *SD* = 11.2).

All participants had normal or corrected-to-normal visual acuity. All subjects were right handed, as assessed by the Edinburgh Handedness Inventory [126]. Each participant provided informed consent to participate in the study, which was conducted in accordance with institutional ethical provisions and the Declaration of Helsinki.

### Experimental design

The experiment involved a three-way repeated-measures factorial design, with group (ASD, control) as a between-participant factor and presentation condition (dynamic, static) and emotion (fear, happiness) as within-participant factors.

### Stimuli

The stimuli were almost identical to those used in a previous fMRI study [40]. The raw materials were grayscale photographs of faces of eight individuals (4 females, 4 males) chosen from a standard set [127] depicting fearful, happy, and neutral expressions. Neutral expressions were adopted as the starting point of the emotional expressions. None of these faces was familiar to any of the participants.

Dynamic expressions were created from photos via computer animation. First, 24 images that increased emotional expression by increments of 4% were created between the neutral (0%) and emotional (100%) expressions using computer-morphing software [128] implemented on a computer operating with Linux. This software was used in several other studies (e.g., [35,36]). Next, to create a moving video clip, a total of 26 images (i.e., one neutral image, 24 intermediate images, and the image of the final emotion) were presented in succession. Each image was presented for 40 ms, and the first and last images were presented for 230 additional ms; thus, each clip lasted for 1500 ms.

The final expressions under the dynamic expression condition were presented as static expressions for 1500 ms.

### Presentation apparatus

The events were controlled by Presentation version 10.0 (Neurobehavioral System) implemented on a Windows computer. The stimuli were projected from a liquid crystal projector (DLA-G150CL, Victor) onto a mirror that was positioned on a scanner in front of the participants. Under these visual conditions, the stimuli subtended a visual angle of about 15.0° vertical × 10.0° horizontal.

### Procedure

The scan session consisted of 12, 20-sec epochs interleaved with 12, 20-sec rest periods in which a blank screen was presented. Each epoch consisted of eight trials, and a total of 96 trials were performed in the scan. Each of the four stimulus conditions (dynamic fear, dynamic happiness, static fear, and static happiness) was presented in different epochs. The order of the epochs was pseudorandomized, and the order of trials within each epoch was randomized.

In each trial, a single individual stimulus was presented for 1500 ms. There was an interval of 1000 ms before the next trial began, during which a fixation point (a picture with a small gray “+” of the same size as the stimulus) was presented on a white background at the center of the screen. The participants were instructed to direct their attention to the center of the screen until the face had disappeared and to specify the sex of the face presented by pressing one of two buttons with the forefinger after the face had disappeared. This task ensured participants’ attention to the stimulus and also prevented idiosyncratic explicit processing for the emotional expression. *Post hoc* debriefing confirmed that the participants were not aware that the purpose of the experiment was unrelated to sex discrimination.

### MRI acquisition

Image scanning was performed on a 3-T scanning system at the ATR Brain Activity Imaging Center (MAGNETOM Trio A, Tim System, Siemens) using a 12-channel array coil without acceleration mode. The functional images consisted of 40 consecutive slices parallel to the anterior–posterior commissure plane covering the whole brain. A T2*-weighted gradient-echo echo planar imaging sequence was used with the following parameters: repetition time (TR) = 2500 ms; echo time (TE) = 30 ms; flip angle (FA) = 90°; field of view (FOV) = 192 × 192 mm; matrix size = 64 × 64; voxel size = 3 × 3 × 4 mm. The order of slices was ascending. After the acquisition of functional images, a T1-weighted high-resolution anatomical image was also obtained using a magnetization-prepared rapid gradient-echo sequence (TR = 2250 ms; TE = 3.06 ms; FA = 9°; inversion time = 900 ms; FOV = 256 × 256 mm; matrix size = 256 × 256; voxel size = 1 × 1 × 1 mm). Elastic pads placed around each side of the participant’s head were used to stabilize head position during functional image acquisition.

### Behavioral data analysis

The percentage and RTs of correct responses were analyzed using three-way repeated-measures ANOVAs with group as a between-participant factor and presentation condition and emotion as within-participant factors. We had no specific predictions for the behavioral data, and hence conducted two-tailed tests. Results were considered statistically significant at *p* < .05.

### Image analysis: Preprocessing

Image preprocessing and regional brain activity analyses were performed using SPM5 (http://www.fil.ion.ucl.ac.uk/spm) implemented in MATLAB version 7 (Mathworks). First, we performed slice-timing correction to correct for the different times needed to acquire slices in functional images. This process was also important to the robustness of the DCM. To correct for head movements, the functional images of each run were then realigned using the first scan as a reference. Data from all participants showed small motion corrections (<2 mm). Subsequently, the T1 anatomical image was co registered to the first scan of the functional images. Next, the co registered T1 anatomical image was normalized to a standard T1 template image as defined by the Montreal Neurological Institute (MNI), which involved linear and non-linear three-dimensional transformations [129,130]. The parameters from this normalization process were then applied to each of the functional images. Finally, these spatially normalized functional images were resample to a voxel size of 2 × 2 × 2 and smoothed with an isotopic Gaussian kernel (8 mm) to improve the signal-to-noise ratio and to compensate for the anatomical variability among participants.

### Image analysis: Regional brain activity analysis

We used random-effects analyses to identify significantly activated voxels at the population level [131]. First, we performed a single-subject analysis [132,133]. The task-related blood-oxygen-level-dependent (BOLD) responses under each condition were modeled with a boxcar function and convoluted with a canonical hemodynamic response function. We used a high-pass filter composed of a discrete cosine basis function with a cut-off period of 128 sec to eliminate the artifactual low-frequency trend. Serial autocorrelation, assuming a first-order autoregressive model, was estimated from the pooled active voxels with a restricted maximum likelihood (ReML) procedure and was used to whiten the data and the design matrix [134]. To reduce the motion-related artifacts, the six realignment parameters of the rigid-body transformation used in the realignment step in the preprocessing were added to the model.

Planned contrast was then performed. The four contrast images of dynamic fear, dynamic happiness, static fear, and static happiness versus rest were entered into the flexible factorial model for each participant and each group, generating a three-way repeated-measures ANOVA to create a random-effect SPM{*T*}. The model included group, presentation condition, and emotion as factors of interest; participant was a factor of no interest (Additional file 1: Figure S1). Based on preliminary analyses, the sex of participants, which showed no significant main effect or interaction in the results, was disregarded in the reported analyses. The non-sphericity correction used in the flexible factorial model corrected for possible differences in variance between the groups due to the unequal sizes of the samples. The same settings were used under the presentation and emotion conditions to correct for uneven variance between levels. The observations that were dependent on presentation and emotion conditions were also corrected. The ensuing covariance components were estimated using ReML and then used to adjust the statistics. This is exactly the same procedure used for serial correlations in single-subject fMRI models. We conducted preliminary analyses to test brain activation under each condition in each group against the resting condition using the same threshold criterion with reported results and found that none of the predicted social brain regions showed significant deactivation. Hence, we did not use any masking procedures.

First, the simple main effect of dynamic versus static presentations was tested for each group. For these analyses, active regions were reported as statistically significant only if they survived the correction for multiple comparisons across the entire brain. Next, our prediction of the interaction between group and presentation condition was tested. For this analysis, about which we had specific predictions, we selected regions of interest (ROIs): the MTG, FG, AMY, MPFC, and IFG. The ROIs were defined as 8-mm-radius spheres centered on the activation foci in the above simple main effect analysis for the control group (cf. [135]). Anatomical specification of the ROIs was conducted using the Talairach Daemon [136] after the transformation of coordinates from the MNI to Talairach systems. All ROIs were confirmed to overlap with the activation foci in previous studies (e.g., [44]). These ROIs were independently examined in an *a priori* manner (cf. [137,138]) by applying small-volume correction [139]. Analyses for this interaction in other brain regions and for other interactions related to the factor of group were conducted in an *a posteriori* manner correcting for the volume of the entire brain. Significantly activated voxels were identified if they reached the extent threshold of *p* < .05 corrected for multiple comparisons, with a height threshold of *p* < .01 (uncorrected). In this setting, the minimum cluster size for the significant extent threshold with the small-volume correction was 58 voxels.

To display the activation patterns across conditions, the parameter estimate under each experimental condition (the *beta* value in the SPM) at the peak voxel of the random-effect analysis was extracted and then averaged across participants.

### Image analysis: DCM

We used DCM [121] to explore how the effective connectivity between brain regions was modulated by dynamic facial expressions. DCM enabled us to draw inferences about the influences that one neural system exerted over another and about how this was affected by the experimental context. Technically, DCM is described as an input–state–output model with multiple inputs and outputs, where inputs are represented by experimental factors determined by the experimental paradigm and outputs are the BOLD signals of all regions. The system dynamics of the interacting brain regions are described by changes in the neural state over time. The modeled neural dynamics are transformed into area-specific BOLD signals by a hemodynamic state model. DCM estimates neural and hemodynamic state parameters with a Bayesian inversion scheme [121]. DCM allowed us to estimate three different types of interactions: (1) intrinsic connections, which represent fixed or baseline connectivity among neural states; (2) modulations of these connections by experimental manipulations; and (3) driving input, which embodies the influences of exogenous input on neural states. In this study, we focused on the modulatory effect of dynamic presentation on the cortical network for facial expression processing.

DCM was performed using SPM8 (http://www.fil.ion.ucl.ac.uk/spm) implemented in MATLAB version 7 (Mathworks). To construct driving and modulatory inputs in our DCM analysis, we remodeled single-subject analyses. The design matrix contained the following three experimental factor-specific regressors: visual input (i.e., all experimental conditions) as the driving input in the DCM; dynamic presentation as the modulatory input; and emotion (fear vs. happiness, which were coded as 1 and -1, respectively). Emotion regressors were included as effects of no interest. Other nuisance regressors (realignment parameters and constant terms), high-pass filters, and serial autocorrelations were at the same settings as for regional brain activity analyses.

To define the cortico–cortical connectivity, we selected three brain regions: the V1 (*x* 22, *y* -84, *z* -4), MTG (*x* 52, *y* -62, *z* 0), and IFG (*x* 56, *y* 28, *z* 10) in the right hemisphere. These ROIs were selected based on our hypothesis described in the Background. The coordinates of the MTG and IFG were defined based on the results of the simple main effect of presentation condition (dynamic vs. static) in the control group. The coordinates of the V1 were derived from the strongest activation focus in the search region of the V1 in response to all stimulus presentations in the control group; this value was defined by the cytoarchitectonic map derived from data on human postmortem brains using the Anatomy Toolbox version 1.5 [140]. The identical activation focus was found in the ASD group using the same procedure to define the V1. The ROIs were restricted to the right hemisphere because some ROIs showed significant activities only in the right hemisphere. ROI time series were extracted for each participant as the first eigenvariate of all voxels within a 3-mm radius around the selected coordinate. These time series were adjusted for the effect of interest and the nuisance effects, high-pass filtered, and corrected for serial correlation.

Next, the hypothesized model was constructed for each participant. The visual input was modeled as the driving input into the V1. The bi-directional (forward and backward) intrinsic connections were constructed between the V1 and MTG and between the MTG and IFG. The modulatory effect of dynamic presentation was modeled to modulate each of these bi-directional connections. Based on the locations of the modulatory effects, we constructed following four models (Figure 3b): the null model, MNS-entrance modulation model, MNS-core modulation model, and full model.

To examine group differences in effective connectivity, we first tested the most appropriate model for each group using random-effect BMS [141]. We used the exceedance probabilities as the evaluation measures based on the belief that a particular model was more likely than any other model given the group data (cf. [142,143]). We next analyzed parameter estimates of the averaged model resulting from BMA. We used the entire model space and computed weighted averages of each model parameter for which the weighting was given by the posterior probability for each model [122,144]. This approach is preferable in a group DCM study in which BMS may indicate a group difference in the model space. To expedite BMA calculation, the low-probability models were excluded from the summation using an Occam's window approach. In this study, Occam’s window was defined using a minimal posterior odds ratio of 1/20 [144]. The modulatory effect parameters were tested with *a priori* interests (cf. [138]) in terms with differences from zero and differences between groups using *t*-tests (one-tailed). The results were deemed statistically significant at *p* < .05.

## Competing interests

The authors declare that they have no competing interests.

## Author contributions

WS, MT, SU and TK designed research; WS, MT, SU and TK obtained the data; WS and TK analyzed the data; and WS, MT, SU and TK wrote the manuscript. All authors read and approved the final manuscript.

## Supplementary Material

Additional file 1 ** Figure S1.** The model for the analysis of regional brain activity.We constructed a three-way repeated-measures ANOVA design including participant as a factor of no interest and group, presentation condition, and emotion as factors of interest. CON = Control; ASD = Autism spectrum disorders; DY = Dynamic; ST = Static; FE = Fear; HA = Happiness. Click here for file
