# Supplementary figures and images for: Impaired social brain network for processing dynamic facial expressions in autism spectrum disorders
Source: BMC Neurosci. 2012 Aug 13;13:99. doi: 10.1186/1471-2202-13-99 (PMC3459703; doi:10.1186/1471-2202-13-99)

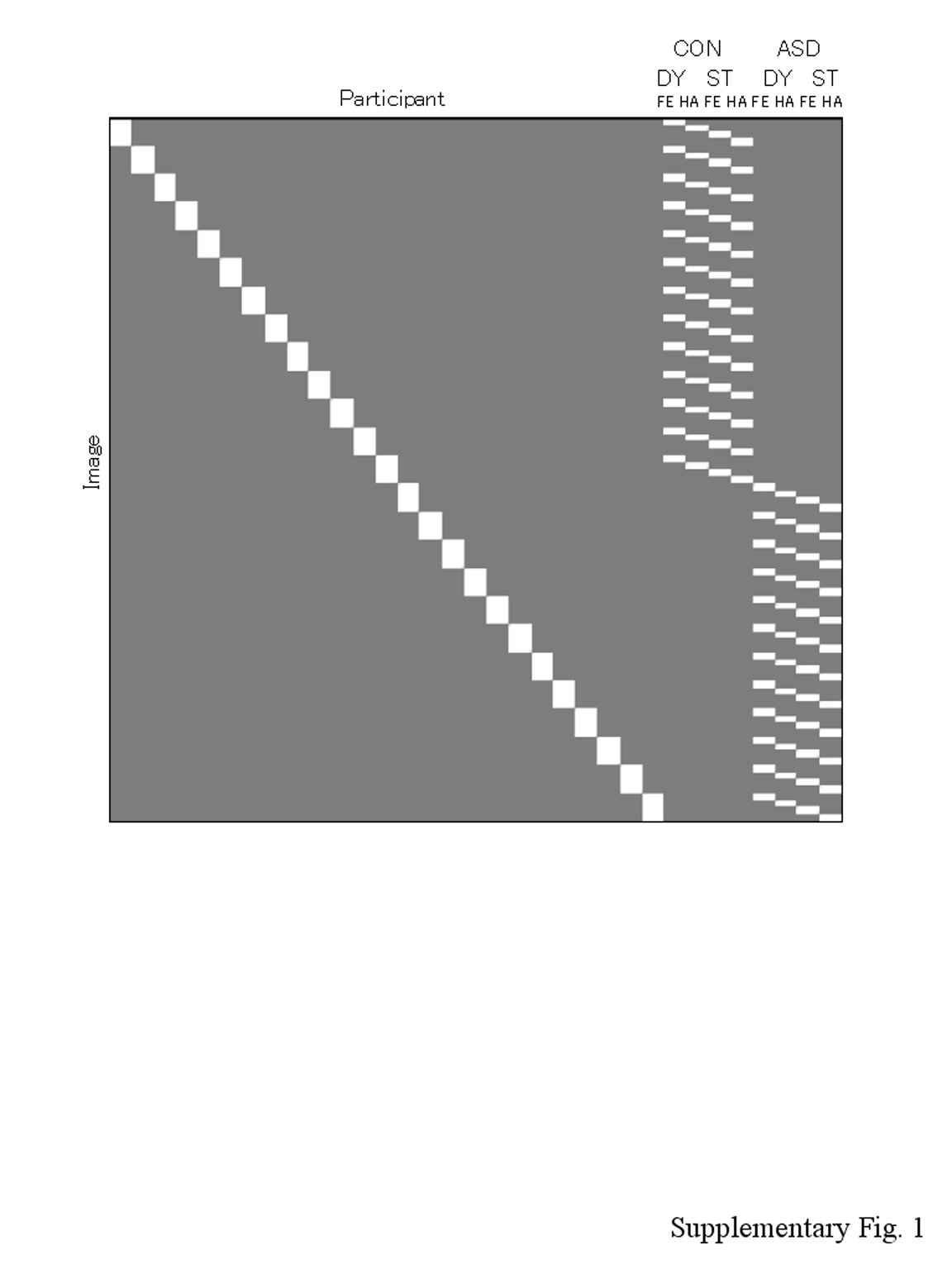

Supplement: Additional file 1 — Figure S1. The model for the analysis of regional brain activity.We constructed a three-way repeated-measures ANOVA design including participant as a factor of no interest and group, presentation condition, and emotion as factors of interest. CON = Control; ASD = Autism spectrum disorders; DY = Dynamic; ST = Static; FE = Fear; HA = Happiness. [file 1471-2202-13-99-S1.tiff]
